# 3D Texture Analysis of the Corpus Callosum in T1-Weighted MR Images of Children with a Traumatic Brain Injury

**DOI:** 10.1007/s10548-026-01188-5

**Published:** 2026-03-16

**Authors:** Jan Novak, Ahmed E. Fetit, Daniel Griffiths-King, Cathy Catroppa, Vicki A. Anderson, Amanda G. Wood

**Affiliations:** 1 Aston Institution of Health and Neurodevelopment, Aston University, Birmingham, B4 7ET UK; 2 Department of Computing, Imperial College London, London, UK; 3 Brain and Mind Research, Clinical Sciences, Murdoch Children’s Research Institute, Melbourne, Australia; 4 Murdoch Children’s Research Institute, Melbourne, Australia; 5 School of Psychology, Faculty of Health, Deakin University, Melbourne Burwood Campus, Geelong, VIC Australia

**Keywords:** Paediatric Traumatic Brain Injury, White Matter, Diffusion Magnetic Resonance Imaging, Texture Analysis

## Abstract

Investigating traumatic brain injuries (TBI) in the developing brain is a challenging task. The superposition of an injury to the normal development trajectory can lead to brain impairments which are not obvious at diagnosis. T1-weighted MRI, acquired routinely post-injury, has the potential to better inform diagnosis, but is limited by qualitative assessment by radiologists. Using T1-weighted volume images, we investigated the use of three-dimensional texture analysis (TA) on regions of the corpus callosum (CC) in children with TBI and typically developing controls (TDCs) in conjunction with analysis of diffusion weighted image (DWI)-derived metrics. Nineteen TDCs and 37 participants with TBI were included in the study. T1 textural metrics were extracted from the splenium, genu and body of the CC and assessed for differences between the groups. Textural skewness was found to be significantly higher in children with TBI than TDCs in the body of the CC (t-test: *p <* 0.004, effect size: g = 0.91) and significant differences were observed in the genu of the CC (grey level co-occurrence matrix and Grey-level run length matrix, *p* < 0.004, effect sizes > 0.6). Non-significant reductions in ADC were found between TBI and TDC groups in the body and the splenium of the CC. Interestingly, no differences were found between TDCs and the TBI sample using FA. The results suggest that TA can potentially be used to assess white matter integrity after paediatric TBI.

**Supplementary Information:**

The online version contains supplementary material available at 10.1007/s10548-026-01188-5.

## Introduction

Traumatic brain injury (TBI) in children can alter the normal course of brain development. Along with external injuries such as lacerations and skull fractures, more subtle damage to the brain can occur such as diffuse axonal injury (DAI). This occurs when the brain moves rapidly within the skull following an impact inducing widespread damage within the brain, including white matter tracts such as the corpus callosum (CC). The CC is a major white matter region connecting the cerebral hemispheres and is highly susceptible to the shearing effects experienced in TBI. The CC has been implicated as important in paediatric TBI where differences have previously been shown between patients and controls and also related to outcome^32^⁠. Neurocognitive outcomes, consistent with disruption to the CC (e.g., slowed speed of processing), are challenging to predict at the individual child level using clinical information and standard clinical Magnetic Resonance Imaging (MRI) protocols(Adams et al. 1989, Meythaler et al. 2001)⁠.

Diagnosis of DAI using standard MRI is challenging and in research contexts accurate detection often requires advanced imaging such as susceptibility-weighted imaging (SWI) or diffusion-weighted imaging (DWI). However, such protocols add significantly to already lengthy scan sessions. Excessive scan times are especially problematic for child studies where participants may not tolerate lengthy scans, resulting in loss of data or systematic bias from motion, particularly for children with brain insult(Raschle et al. 2012)⁠. There is significant advantage in utilising clinically acquired scans, such as T1- and T2-weighted images to produce more precise quantitative measures that can feed into clinical pathways.

Texture analysis (TA) of Magnetic Resonance Imaging (MRI) is an automated method and can be likened to the role of a radiologist in that perceptions of patterns within an image can be used to aid interpretation of clinical imaging(Bharati et al. 2004)⁠. Implementation of automated or semi-automated techniques into the diagnostic pipelines has the potential to reduce inherent variability(Anderson et al. 1989, Gorovitz and MacIntyre 1976)⁠ incurred by human interpretation. For example, Holli et al. investigated hemispherical asymmetry in TBI patients using texture analysis^8^⁠ and found hemispherical textural differences between patients with mild TBI patients whereas in contrast there was minimal variation in healthy controls.

TA is a broad umbrella term for the analysis of grey levels within an image which encompasses the spectral features, pixel interrelationships and grey level (relative intensity) patterns. Initially used for photography, TA has been introduced to medical imaging and while not yet broadly adopted, has shown interesting findings, mainly in the field of oncology(Fetit et al. 2014, Fetit et al. 2018, Fetit et al. 2015, Skogen et al. 2016, Kim et al. 2017)⁠. The first order statistics, variance, skewness are analogous to histogram metrics well described throughout quantitative science. Grey-Level Co-Occurrence matrix measures are a group of second order statistics which pick out patterns from pairs of pixels in images. These include Homogeneity and Entropy which measure the smoothness and coarseness respectively. Contrast describes the difference in in intensity between adjacent pixels. Entropy measures the randomness within the image and Autocorrelation and Correlation both quantify the spatial dependencies of the grey levels within the image. The grey-level run-length matrices investigate the intensity of consecutive pixels, providing information on directional image homogeneity. Other examples of TA in the medical imaging include diagnostic classifiers for paediatric brain tumours(Fetit et al. 2014, Fetit et al. 2018)⁠ and investigating medication overuse headaches related to textural features in grey matter(Chen et al. 2017).

In the current work we produced three-dimensional (3D) textural features from CC sub-regions acquired from conventional T1-weighted images for the TBI and TDC groups. We tested the textural features on a group-wise basis to see if they captured any discriminatory patterns. We were not looking to classify between TBI and TDC groups but to explore brain imaging differences between injured and non-injured children. We hypothesised that textural features obtained from T1-weighted images would show more disorder in white matter appearance following a traumatic brain injury. We also investigated Fractional Anisotropy (FA) and Apparent Diffusion Coefficient (ADC) metrics derived from DWI for reference. Although the literature is conflicted for these metrics, as the physiological underpinnings are currently better understood, they provided a reference point for the textural features.

## Materials and Methods

### Participants

Analysis for the current study was made possible through a material transfer agreement between the Murdoch Children’s Research Institute and Aston University. Ethical approval was granted from Aston University as a site for secondary analysis of neuroimaging data which represented a subset of a prospective, longitudinal study of outcomes from paediatric TBI (Prevention and Treatment of Social Problems Following TBI in Children and Adolescents). The current sample consisted of a total of 157 children recruited. Further details of the study including details of the recruitment strategy have recently been published elsewhere(Anderson et al. 2017, Catroppa et al. 2017)⁠. The data for this experiment are a subset of a larger dataset involving children aged 5 to 16 who experienced a TBI. Eligibility criteria included: (i) age 5 to 16 at the time of injury, (ii) documented closed-head injury with at least two post-concussive symptoms (e.g., headaches, dizziness, irritability), (iii) sufficient medical records to assess injury severity (Glasgow Coma Scale (GCS), neurological and radiological findings), (iv) no prior neurological or neurodevelopmental disorders, non-accidental injuries, or previous TBI, and (v) English proficiency. TD controls were recruited from the community and had to meet criteria i), iv), and v).

Injury type was broad including motor vehicle accident (patient as passenger in car, pedestrian or on a bike), or fall/blow (either from stationary for example falling tree, fall from roof or moving for example trampolining). Socioeconomic Status was indexed using highest caregiver’s AUSEI06 ranking. Median highest AUSEI06 ranking for Controls = 81.3, whilst TBI patients = 71.6. Mean highest AUSEI06 ranking for Controls = 77.9, whilst TBI patients = 66.5. Mean socioeconomic status was significantly higher in the control versus patient group (t(51) = 2.743, CI95 [3.054 19.715], *p* = 0.008) (Welch Two Sample T-Test). Age at MRI did not differ between patients and controls (t(25) = 0.740, CI95 [-0.970 2.058], *p* = 0.466^a^) (Welch Two Sample T-Test) nor did the proportion of males to females between groups (χ^2^(1) = 0.709, *p* = 0.400^b^) (Pearson’s Chi Squared test with Yates Continuity Correction).

Of the participants recruited, 107 had a TBI and 36 TDCs had MRI scans acquired. MRI scans were acquired in the acute post-injury period for patients. T1-weighted images were qualitatively checked by JN and DK for low SNR; additionally, any images with motion artefacts were removed from the study. DWI scans with significant motion or distortion artefact were also removed from the dataset. The scans with the significant artefact were removed prior to any pre-processing stage described below. The QA procedure was based on the paper by Backhausen et al. (Backhausen et al. 2016). ⁠. Heterogeneity within the study protocol resulted in both b = 1000 and b = 2000 being implemented for the DWI scans. ADC is dependent on b-value so scans with b = 2000 were exclusively used. Following the aforementioned quality assessment and data selection procedure the cohort consisted of the subjects summarised in Table 1. There was no significant difference between the age of the participants.

**Table 1:** A table showing the details of the final study sample

Total Number	TBI	TDC
	37	19
Mean Age at Scan /Years	10.7	11.5
Mean Age at Injury/Years	10.4	N/A
Age Range /Years	6.1–14.1	6.7–15.9
Mean Injury-Scan Interval /Days	37	N / A
Range Injury-Scan Interval /Days	16–73	N / A
Male	27	11
Female	10	8
Mild Injury	24	N / A
Mild-Complex Injury	3	N / A
Moderate Injury	6	N / A
Severe Injury	4	N / A

### Image acquisition

MR-Images were acquired for the TBI group acutely after injury (16–73 days post-injury). Acquisition was carried out at 3T, as a part of an existing research protocol, on a Siemens Trio scanner (Siemens Medical Systems, Erlangen, Germany) using a 32-channel matrix head coil. 3D T1-weighted images were acquired in the sagittal plane using an MPRAGE sequence. TR = 1900; TE = 2.15 ms; IR prep = 900 ms; parallel imaging factor = 2 (GRAPPA); flip angle = 9 degrees; BW 200 Hz/Px; 176 slices; resolution 1 × 0.5 × 0.5 mm. DWI was acquired using 60 directions at b = 2000 s/mm2; TR = 9300; TE = 104; voxel size 2 × 2 × 2 mm^2^ and matrix size of 128 × 128 × 128.

### Image Analysis

#### Image registration and Segmentation

The raw image data were transformed into a common space using the JHU white matter atlas(Ho and Black 2010, Wakana et al. 2007, Hua et al. 2008)⁠. This was done via an initial linear registration using FSL (version 5.0) FLIRT(Hua et al. 2008, Jenkinson et al. 2002) followed by a non-linear warp using FSL FNIRT. To ensure that only white matter was included in the analysed regions of interest the white matter atlas needed to be trimmed slightly. For this, a binary white matter population template was created from the control cases (*n* = 19). This was done via a FSL FIRST(Patenaude et al. 2011)⁠ segmentation for registered images. The white matter masks were then averaged to create the template. This population template was used to trim the JHU parcellations which were also manually checked for the inclusion of erroneous tissue. The JHU labels from the atlas for the corpus callosum (body, genu and splenium) were used to extract the pixels from the regions of interest for both imaging modalities for further analysis.

### DWI analysis

The MR data were pre-processed using a combination of FSL (version 5.0)(Smith et al. 2004) ⁠ and MRTRIX3 (www.mrtrix.org)(Tournier et al. 2019) including noise reduction, eddy current, motion, and bias field correction(Smith et al. 2004, Veraart et al. 2016, Andersson and Sotiropoulos 2016, Zhang et al. 2001)⁠. Following the pre-processing a tensor image was produced using the dwi2tensor command in MRTRIX3. From this tensor image, FA and ADC maps were generated using the tensor2metric command(Basser et al. 1994)⁠. The mean ADC and FA values for the regions of interest were calculated using the MRTRIX3 mrcalc function.

### T1 Texture analysis

3D texture analysis was carried out on normalized(Collewet et al. 2004)⁠, T1 images using MATLAB (version 2017a, The MathWorks, Inc., Natick, Massachusetts, United States) and the Radiomics toolbox(Vallières et al. 2015). The calculated texture analysis metrics are summarised in Table 2. The run length metrics were averaged over all directions for the final result.

**Table 2:** A table showing the metrics extracted from the T1-weighted images following texture analysis

Texture Analysis Method	Metrics
First Order Statistics	Variance, Skewness, Kurtosis
Grey-level co-occurrence matrix	Energy, Contrast, Entropy, Homogeneity, Correlation, Autocorellation
Grey-level run length matrix	Long Run Low-Grey Run Emphasis (LRLGRE), Long Run High-Grey Run Emphasis (LRHGRE), Grey Level Variance (GLV), Run Level Variance (RLV)

### Statistics

The subsequent statistical analysis was performed in SPSS (version 23, IBM). The mean ADC and FA values were assessed between patients and controls via a two-tailed independent t-test for unequal variance. A Bonferroni-corrected *p* < 0.025 was used for as the boundary of significance for a multiple comparison correction across the two metrics. TA metrics were also tested for differences via a two-tailed t-test for unequal variance. For values that were significantly different a one-way ANCOVA with patient vs. control as the fixed factor and SES as a covariate, run separately for each imaging variable. A Bonferroni-corrected *p* < 0.004 to for a multiple comparison correction across the 12 metrics. The effect size was Hedges’ g and was reported for both TA and diffusion metrics. This provided an effect size relative to the size of each sample, ideal for our cohort where the TBI and TDC groups were different sizes.

The violin plots produced for the visualisation of difference in metrics were produced in R (version 3.5.0) and ggplot (version 3.1.0).

## Results

The figure in the supplementary material shows the relationship between outcome measures, demographic variables and texture analysis. Although strong correlations were observed between the textural features, nothing substantial was detected between the outcome measures or demographic variables. There were no significant group differences for age, t(26) = -0.64, *p* = 0.530.

**Fig. 1:**
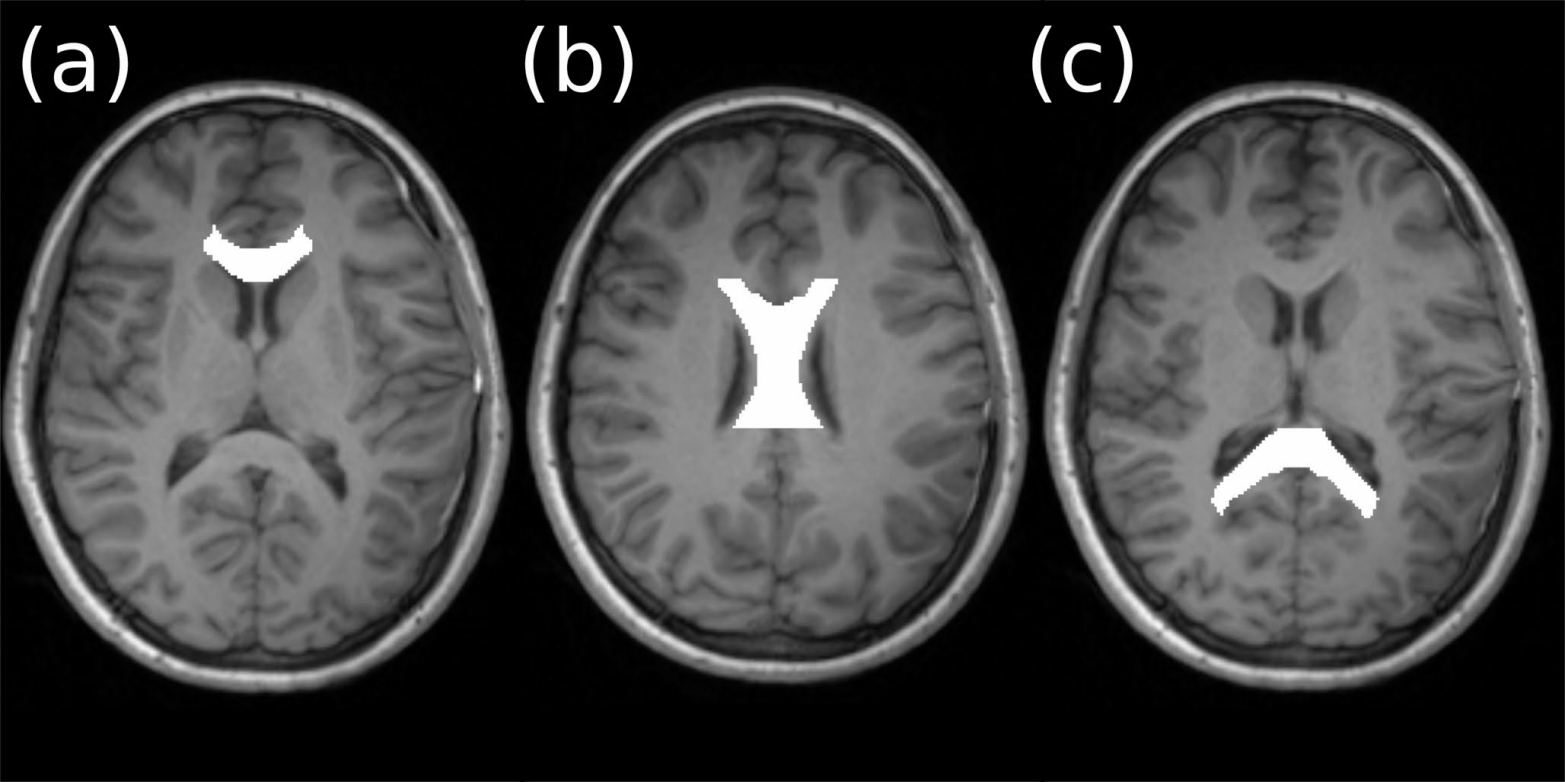
3D T1-weighted images displayed in the axial plane. The regions of interest displayed on the images represent the parcellations of the corpus callosum used in the analysis. (**a**) genu, (**b**) body and (**c**) splenium

The three regions of the CC, Genu, Body and Splenium are illustrated in Fig. 1).

**Table 3:**
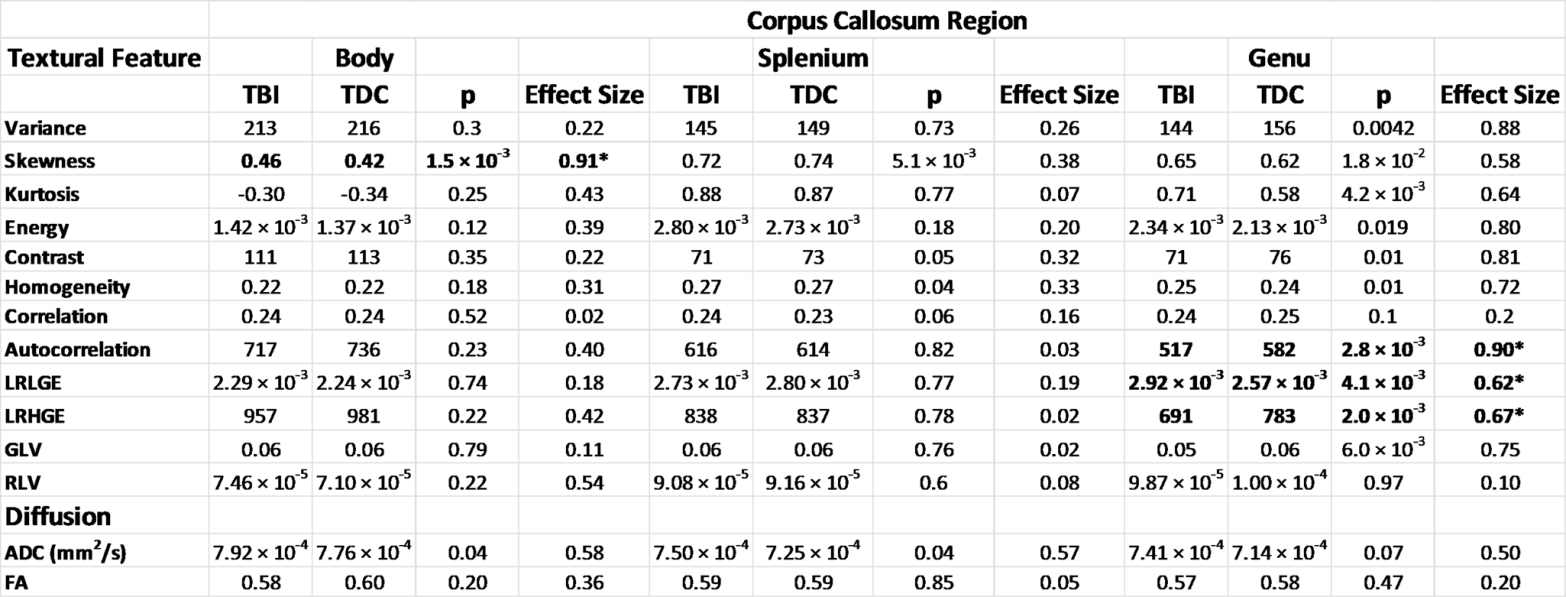
A table showing the texture analysis and diffusion metrics extracted from T1-weighted images from Body, Genu and Splenium of the Corpus Callosum. The effect sizes presented are Hedges’ g and the p-value is derived from a two-tailed t-test for unequal variance. A Bonferroni-corrected threshold for significance for the TA metrics was p  < 0.025 and p  < 0.025 for the DWI metrics, indicated by *

Both TA and DWI metrics in the CC were compared using T-test and hedges’ g effect sizes (Table 3). No significant differences were observed for the TBI group compared to TDCs following correction for multiple comparison. ADC was non-significantly higher in the TBI group compared to TDCs in the genu. No significant group differences in FA were found anywhere in the corpus callosum.

Skewness, a TA histogram metric, was significantly increased in the TBI group compared to the TDCs in the body of the CC (g = 0.91). No significant differences were observed for any TA features in the splenium. In the genu, the grey-level co-occurrence matrix (GLCM) metric Autocorrelation was found to be significantly increased in TDCs compared to the TBI group (g = 0.90). Long run low grey emphasis (LRLGE) was significantly increased and long run high grey emphasis (LRHGE) was significantly decreased in the TBI group compared to TDCs (g = 0.62 and 0.67 respectively). LRLGE and LRHGE were strongly negatively correlated in the genu (Pearson σ = 0.86).

**Fig. 2:**
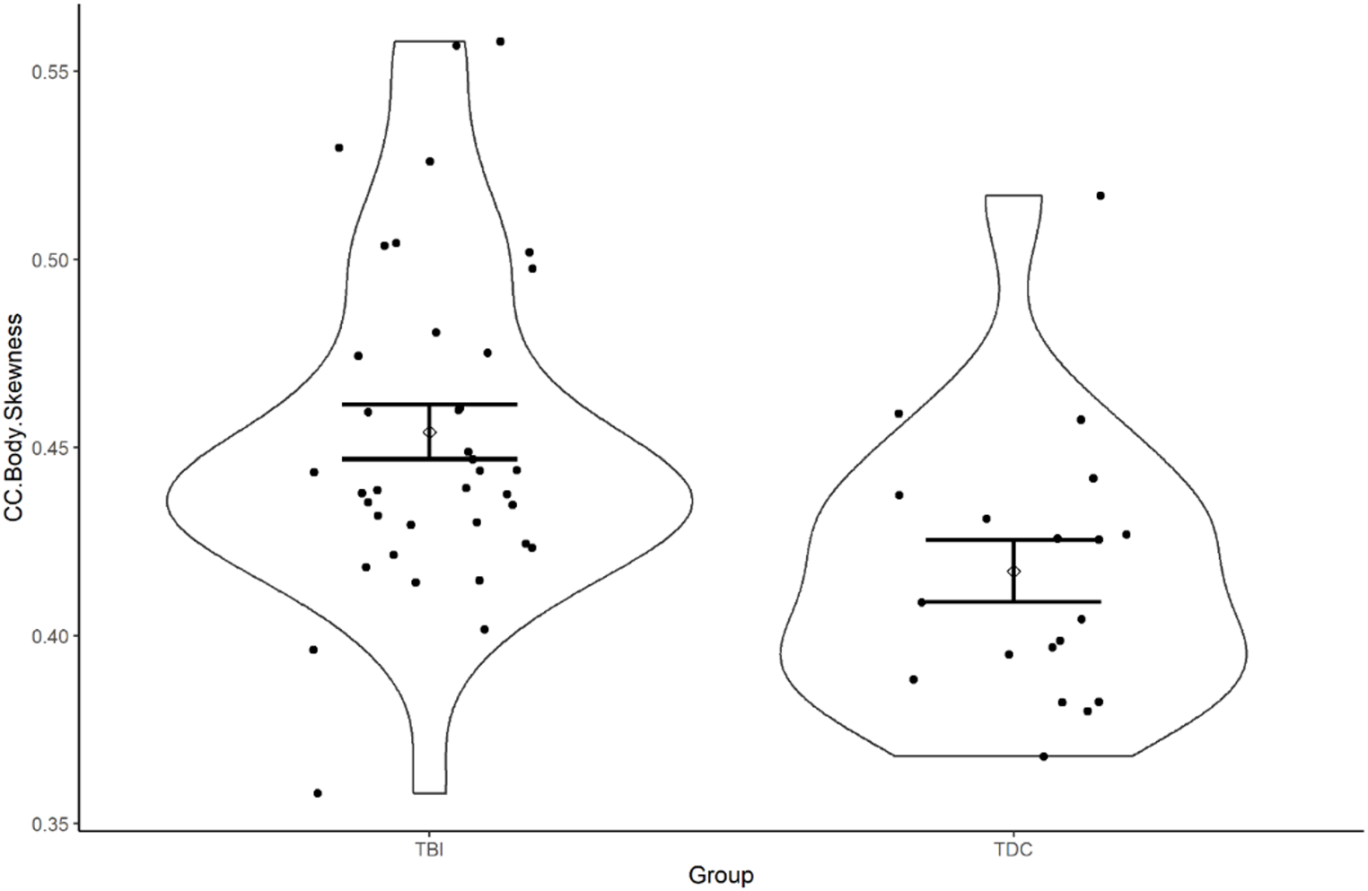
shows the distribution of a selected texture analysis metric using violin plots for TDC and TBI groups. The violin plots show the difference in the Skewness for the Body of the CC. A significant difference was observed and also the spread of the data and the 95% confidence intervals are demonstrated

## Discussion

The purpose of this study was to investigate textural features extracted from 3D T1-weighted MRI images which are routinely acquired for children with TBI and compare them with TDCs. The CC has been implicated as important in paediatric TBI, where differences have previously been shown between patients and controls and also related to outcome(Dennis et al. 2017)⁠. We showed that, in certain regions of the CC, the textural appearance of white matter in T1-weighted images is significantly different in patients with a TBI. Although T1 is not the modality of choice for identification of damage(Eierud et al. 2014)⁠ following a TBI, it is routinely acquired as part of routine clinical practice and provides an opportunity to tease out potential quantitative biomarkers.

A higher skewness found in the body of the CC in the TBI group compared to controls indicated a divergent distribution of image intensities between the two groups. This can potentially be related to the underlying microstructure of the imaged tissue where non-uniformity of the white matter could be caused by damage from a traumatic brain injury(Klimaj et al. 2012)⁠. The lower autocorrelation observed for the patients in the genu reflects a lower likelihood of a pair of grey levels will co-occur. This means that the likelihood that a pair of adjacent voxels with identical intensity will be observed multiple times within a region. This higher-order statistic indicated decreased homogeneity for the TBI group within the genu which can potentially be due to incurred damage. The autocorrelation may be picking up white matter abnormality, which is not obvious to the naked eye, or even white matter abnormality that can be identified qualitatively by a radiologist. The LRLGE and LRHGE were strongly negatively correlated so can effectively be treated as a single metric for interpretation. Both metrics were significantly different for patients and controls suggesting that the long-range coherence of grey levels in the genu has been affected by the TBI. The significance of the high or the low grey value aspect of this result would be an over-interpretation. In practice, more could be learned from comparison of TA with existing imaging modalities such as SWI, DWI and CT. Alterations in white matter image properties in the CC have been documented for advanced imaging modalities such as DWI(Dennis et al. 2015) and SWI(Shenton et al. 2012)⁠. However, the mechanism behind T1 MRI acquisition make it unlikely that the presence of micro bleeds would impact image intensity, however it may be possible to pick up long-term effects of haemorrhage.

Differences have previously been observed using TA in adult TBI populations where intra-patient hemispherical TA differences were found to be greater than those observed for controls(Holli et al. 2010)⁠. For example, Holli et al.(Holli et al. 2010) ⁠ identified an association between hemispherical asymmetry using TA which was found to be related to verbal memory. Comparison to neuropsychological outcomes may also provide information of subtle brain damage in the absence of histopathological correlates.

The increase in ADC for TBI compared to TDC groups (which did not survive the correction for multiple comparisons) observed in the body and the splenium of the CC showed that there are microstructural changes to the white matter which may be related to the TA metrics. Increases in ADC have been reported in children following TBI and have been related to a reduction in cellularity and an increase in water content(Dennis et al. 2017).

There is an ongoing debate in the literature on the utility of FA and ADC (or MD) in the acute and post-acute phases of TBI(Dennis et al. 2017)⁠. The injury-to-MRI time frame for the scans in this study reside in the period between acute and post-acute so direct comparison of values and effects are challenging.

The non-significant differences in ADC observed in the current study are different to what was reported in the literature in a relatively small study of concussed patients (*n* = 12), where a decrease in ADC was observed(García Santos and Ordóñez 2008)⁠. ADC is known to be a non-specific biomarker when compared cellular architecture in both healthy and damaged/diseased tissue(Skogen et al. 2016). Reduced ADC and no TA differences detected in the splenium suggested that the two variables are independent and have differing underlying morphologies. This is further corroborated by extensive differences TA observed in the genu and no ADC changes. This is perhaps unsurprising as for many pathologies DWI and T1 are used as complimentary modalities.

Interestingly, no FA differences were observed between the TBI and control groups. FA has generally been reported to be higher in the CC in the acute and post-acute phase(Virji-Babul et al. 2013)⁠. This trend may potentially reverse, however, in the chronic phase where FA is generally observed to be reduced(Dennis et al. 2017). It must be mentioned that there are only a limited number of studies available in the literature, thereby making direct comparisons challenging.

### Limitations

The cohort size for the study is small, reducing the statistical power for the analysis, especially given the number of multiple comparisons. The small dataset was partly due to rigorous QA and removal of noisy data which is not clinically-realistic and this needs further exploration.

Further metrics can be extracted from the DWI data including tractography metrics, axial and radial diffusivity, and more novel measures such as fibre density and fibre cross-section which could be compared to the TA metrics but were beyond the scope of this study. The inherent problem with texture analysis is the poorly understood semantic relationship between the extracted metrics and the underlying biology. Correlation with a broader range of imaging modalities may aid in interpretation of results.

It is important to note, to our knowledge, there is no understanding of how textural features within the white matter change as a function of age. This requires further systematic exploration. The clinical question of the textural differences between children with TBI and typically developing children is an interesting one but the key clinical relevance resides in accurate diagnosis and prognosis for children and their families.

## Conclusions

We showed that 3D texture analysis of conventional MRI can be performed on white matter in a paediatric TBI cohort, namely the CC to assess differences between children with TBI and those developing typically. We observed significant group differences in the texture between the genu and the body of the CC. We have shown it has the potential to be a complimentary technique which can potentially be used in tandem to assess white matter morphometry. This is a technique that could potentially be applied to cohorts with a wide range of white matter disorders. Given that some TBI patients suffer from neurocognitive disorders texture analysis of white matter may provide an additional information in identifying those patients who require clinical follow up.

## Supplementary Information

Below is the link to the electronic supplementary material.

Supplementary Material 1

## Data Availability

No datasets were generated or analysed during the current study.

## Abbreviations

TA: Texture Analysis
MRI: Magnetic Resonance Imaging
DWI: Diffusion Weighted Imaging
FA: Fractional Anisotropy
ADC: Apparent Diffusion Coefficient
MD: Mean Diffusivity
TBI: Traumatic Brain Injury
TDC: Typically Developing Control
JHU: John Hopkins University
CC: Corpus Callosum
GLCM: Grey-level co-ocurrence matrix
GLRLM: Grey-level run length matrix
SRE: Short Run Emphasis
LRE: Long Run Emphasis
GLN: Grey-Level Nonuniformity
RLN: Run-Level Nonuniformity
RP: Run Percentage
LGRE: Low-Grey Run Emphasis
HGRE: High-Grey Run Emphasis
SRLGRE: Short Run Low-Grey Run Emphasis
SRHGRE: Short Run High-Grey Run Emphasis
LRLGRE: Long Run Low-Grey Run Emphasis
LRHGRE: Long Run High-Grey Run Emphasis
GLV: Grey Level Variance
RLV: Run Level Variance
