# Supplementary material for: 3D Texture Analysis of the Corpus Callosum in T1-Weighted MR Images of Children with a Traumatic Brain Injury
Source: Brain Topogr. 2026 Mar 16;39(3):32. doi: 10.1007/s10548-026-01188-5 (PMC12992439; doi:10.1007/s10548-026-01188-5)
Supplement: Supplementary file 1 — Supplementary Material 1 [file 10548_2026_1188_MOESM1_ESM.docx]

Supplementary material


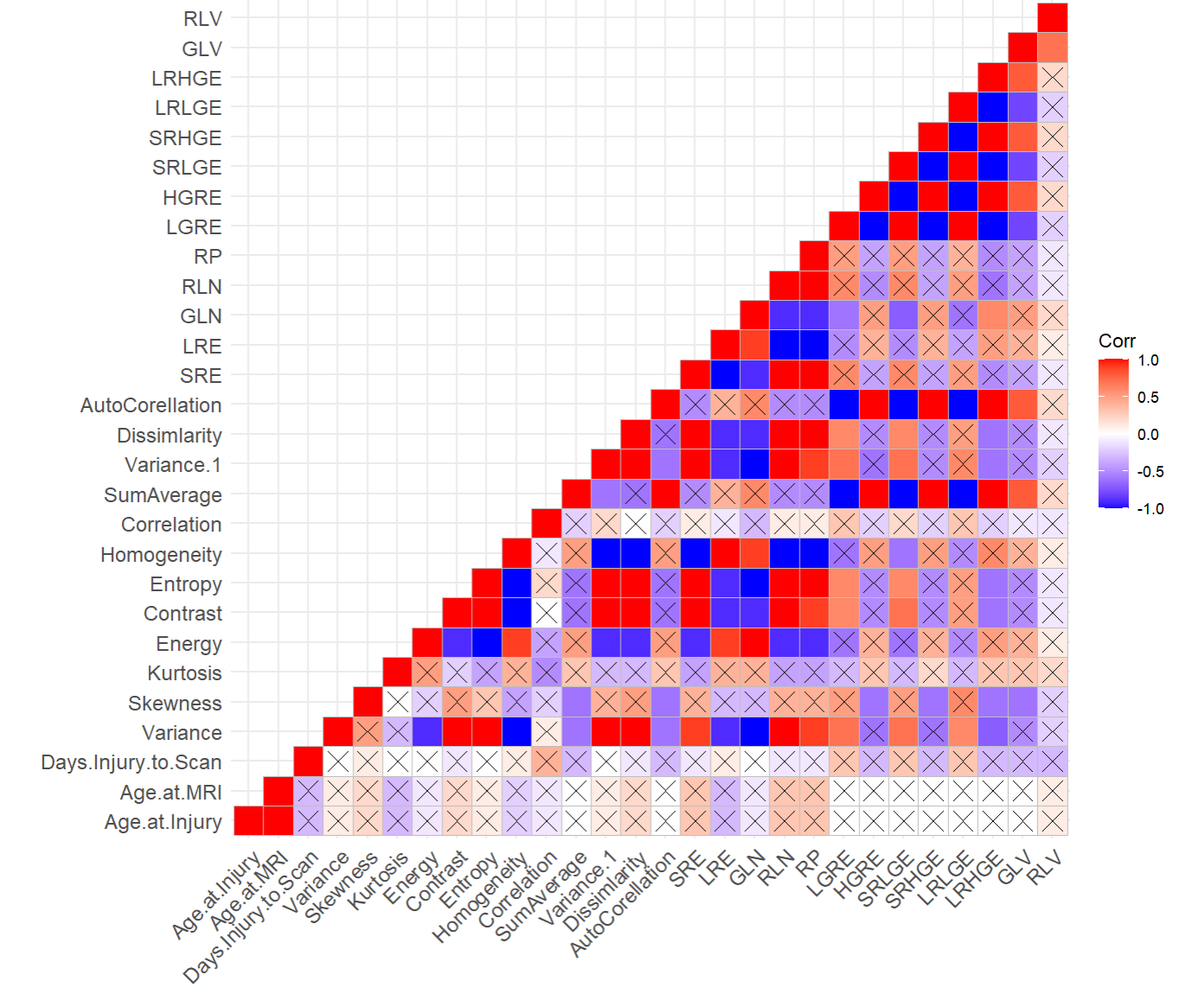


Supplementary Figure 1) A Correlation matrix (Pearson’s correlations) between texture metrics and Injury variables (age, age at injury, time between injury and MRI) in the patient cohort. Strength of correlation indicated by colour, whilst crosses indicate correlations that did not meet threshold for significance (Critical α (Bonferonni Corrected) = 0.05/378 to correct for multiple comparisons within the correlation matrix). Results show no apparent relationship between Texture and either age at scan or injury, or days between injury and scan. Plotted using R package ggcorrplot. (v0.1.4.1).
